# Gray-level invariant Haralick texture features

**DOI:** 10.1371/journal.pone.0212110

**Published:** 2019-02-22

**Authors:** Tommy Löfstedt, Patrik Brynolfsson, Thomas Asklund, Tufve Nyholm, Anders Garpebring

**Affiliations:** Department of Radiation sciences, Umeå University, Umeå, Sweden; INSERM, FRANCE

## Abstract

Haralick texture features are common texture descriptors in image analysis. To compute the Haralick features, the image gray-levels are reduced, a process called quantization. The resulting features depend heavily on the quantization step, so Haralick features are not reproducible unless the same quantization is performed. The aim of this work was to develop Haralick features that are invariant to the number of quantization gray-levels. By redefining the gray-level co-occurrence matrix (GLCM) as a discretized probability density function, it becomes asymptotically invariant to the quantization. The invariant and original features were compared using logistic regression classification to separate two classes based on the texture features. Classifiers trained on the invariant features showed higher accuracies, and had similar performance when training and test images had very different quantizations. In conclusion, using the invariant Haralick features, an image pattern will give the same texture feature values independent of image quantization.

## Introduction

The easiest and most intuitive image features for most applications in image analysis are first order statistics computed from histograms of the gray-level values in images, like their mean, variance, skewness and kurtosis. Such features involve the values of individual pixels, but ignore the spatial interaction between pixels. Histogram features do not reflect objects or patterns in the image, only the distribution of gray-levels. This inability makes first order statistics a blunt tool for quantifying changes in images, or any change in the spatial distribution of gray values.

Most texture analysis methods use higher-order statistics, and consider the relation between two or more pixels at a time. Methods such as local binary patterns [1], wavelets [2] and Gabor filters [3] can be used to assess texture in images. Haralick *et al*. [4] proposed using a gray-level co-occurrence matrix (GLCM) as a method of quantifying the spatial relation of neighboring pixels in an image. Haralick texture features, computed from the GLCM, are widely used due to their simplicity and intuitive interpretations, and have successfully been applied in *e.g*. in the analysis of skin texture [5], in land-use and forest-type classification [6], in automatic pollen detection [7], in fabric defect detection [8], in plant leaf classification [9], in cutting tool condition monitoring [10], and electrophoresis analysis [11]. In recent years there has been a rapid increase in the application of Haralick features in medical image analysis, *e.g*. in the analysis of ultrasound and MRI images of the liver [12, 13], the heart [14], X-ray mammography [15, 16], MRI images in the study of breast cancer [17, 18], prostate cancer [19–21] and brain cancer [22–24]. It is also used in radiomics [25, 26], which is an emerging technique where a large number of quantitative features are extracted from medical images, and used to build models predicting *e.g*. tumor phenotype [27], survival [28, 29] and tumor classification [30].

When creating a GLCM from an image or a region of interest (ROI), the image bit depth, *i.e*. the number of gray-levels, may be reduced in a process called quantization. It is, however, not straight-forward to set an appropriate bit depth, and many projects have been looking at the optimal bit depths for various applications. [28, 31–35] The optimal bit depth depends on the size of the image or ROI, image noise [36] and, of course, the content of the image [33]. To complicate things further, the values of the Haralick features depend strongly on the bit depth and the choice of maximum and minimum values used in the quantization [34, 37]. Clausi *et al*. [38] proposed a method for normalizing two features with respect to the quantization gray-levels to improve classification, however this method is not generalizable to all Haralick features. Shafiq-ul-Hassan *et al*. [39] proposes another method for scaling a selection of the Haralick features to make them less sensitive to the quantization gray-levels. The presented approach is empirical, and no general rule for feature normalization is presented. Currently, most Haralick feature values cannot be compared between analyses using different numbers of gray-levels, even when they are computed on images depicting the same texture. This means that Haralick features cannot be used to create general models that can be applied regardless of image size or noise levels, and are not reproducible unless the same quantization is performed.

The aim of this work is to propose a modified set of Haralick texture features that are asymptotically invariant to the image quantization, while preserving most of the interpretations of the original features. The modified features allow statistical models to be constructed from images with varying number of gray-levels, or to apply a model trained on one number of gray-levels to image data using a different number of gray-levels.

## Theory

**The first step** in texture analysis using the Haralick features is to map the gray-levels in the original image with size *M* × *K*, **I**^**′**^, from the range [*a*, *b*], to a quantized image, **I**, in the range [1, *N*], that has the desired number of gray-levels, *N*. We represent this step with a quantization function

$$\begin{array}{c} \varphi :{\left[a,b\right]}^{M\times K}\to {\left[1,N\right]}^{M\times K}, \end{array}$$ (1)

that returns the quantized image

$$\begin{array}{c} I=\varphi (I^{\prime }). \end{array}$$ (2)

In this work we have set *φ* to be an affine function, but there is no constraint on this function for the purpose of this work.

**The second step** is to construct the non-normalized GLCM, $X\in N^{N\times N}$, by counting the number of times each pair of gray-levels occurs as neighbors the image, **I**, or in an arbitrary region in **I**. The neighbor of a pixel is defined by a displacement vector, *δ* = (*d*_*x*_, *d*_*y*_), in two dimensions, where $d_{x},d_{y}\in Z$ represent the displacement in *x* and *y* in units of pixels. Each element, *X*(*i*, *j*), in the GLCM is computed as

$$\begin{array}{c} X\left(i,j\right)=\sum_{m=1}^{M}\sum_{k=1}^{K}\{\begin{array}{cc} 1, & \text{if}\;I\left(m,k\right)=i\;\text{and}\;I\left(m+d_{x},k+d_{y}\right)=j,\\ 0, & \text{otherwise}, \end{array} \end{array}$$ (3)

where *I*(*m*, *k*) is element *m*, *k* in the quantized image **I**. Simply put, the element *X*(*i*, *j*) of the non-normalized GLCM counts how many times gray values *i* and *j* occur as neighbors in **I**, where *i*, *j* ∈ [1, *N*]. It is possible to create a GLCM with several displacement vectors, *e.g*.

$$\begin{array}{c} \begin{array}{cccc} \delta_{0^{{}^{\circ}}} & =(1,0) & \delta_{180^{{}^{\circ}}} & =(-1,0)\\ \delta_{45^{{}^{\circ}}} & =(1,1) & \delta_{225^{{}^{\circ}}} & =(-1,-1)\\ \delta_{90^{{}^{\circ}}} & =(0,1) & \delta_{270^{{}^{\circ}}} & =(0,-1)\\ \delta_{135^{{}^{\circ}}} & =(-1,1) & \delta_{315^{{}^{\circ}}} & =(1,-1), \end{array} \end{array}$$ (4)

for the eight immediate neighbors of a pixel, in two dimensions. This can be extended to three dimensions, in which case 26 displacement vectors would be required. A GLCM created using all of these displacement vectors, *e.g*. by summation, is said to be direction invariant. If displacement vectors with opposite directions are used to construct the GLCM, the GLCM will be symmetric. Symmetric GLCMs are semi-direction invariant, they represent relations between pixels in *e.g*. vertical or horizontal directions instead of up or down, left or right. A semi-direction invariant GLCM can also be created by adding the transpose of a GLCM created by one displacement vector to itself. This means that only one of the columns in Eq 4 needs to be calculated to obtain a direction invariant GLCM in two dimensions, or 13 displacement vectors for GLCMs constructed from neighbors in three dimensions.

**The third step** is to construct a normalized GLCM, $P\in Q^{N\times N}$, where each element represents the estimated probability of each combination of pairs of neighboring gray-levels in the image. The normalized GLCM is computed as

$$\begin{array}{c} P=\frac{X}{\sum_{i=1}^{N}\sum_{j=1}^{N}X\left(i,j\right)}. \end{array}$$ (5)

The normalized GLCM can be interpreted as a probability mass function of the gray-level pairs in the image.

**The fourth step** is to compute the texture features from the normalized GLCM. The Haralick texture features are functions of the elements and their corresponding indices in the normalized GLCM, and can be written in a general form as

$$\begin{array}{c} f=\sum_{i=a}^{A}\sum_{j=b}^{B}\phi (i,j,g(P))\psi (p(i,j)), \end{array}$$ (6)

where *ψ* is a function of an element of **P**, **g** is a vector-valued function of **P**, and *ϕ* is a function of the indices and **g**. Examples of **g** are found in in Table 1. For instance when computing *Correlation* (see Table 2), we have

$$\begin{array}{cccc} a & =b=1\;, & A & =B=N\;,\\ g(P) & =(\mu_{x},\mu_{y},\sigma_{x},\sigma_{y})\;, & \psi (p(i,j)) & =p(i,j)\;,\\ \phi (i,j,g(P)) & =(\frac{i-\mu_{x}}{\sigma_{x}})(\frac{j-\mu_{y}}{\sigma_{y}})\;, & f & =\sum_{i=1}^{N}\sum_{j=1}^{N}(\frac{i-\mu_{x}}{\sigma_{x}})(\frac{j-\mu_{y}}{\sigma_{y}})p\left(i,j\right)\;. \end{array}$$

**Table 1:** Variables and notation used to compute the Haralick texture features. The two left-most columns show the original definitions, and the two right-most columns show the modifications needed to make the feature invariant to the number of gray-levels.

Notation	Definition	Mod. Notation	Definition
*x*(*i*, *j*)	Element *i*, *j* in the unnormalized GLCM		
*N*	Number of gray-levels		
		Δ	$\frac{1}{N}$
		Δ_*x*+*y*_	$\frac{1}{2N-1}$
		Δ_*ij*_	$\frac{1}{N^{2}}$
*p*(*i*, *j*)	$\frac{x(i,j)}{\sum_{i=1}^{N}\sum_{j=1}^{N}x\left(i,j\right)}$	$\tilde{p}\left(i,j\right)$	$\frac{x(i,j)}{\sum_{i=1}^{N}\sum_{j=1}^{N}x\left(i,j\right)\;\Delta_{ij}}$
*p*_*x*_(*i*)	$\sum_{j=1}^{N}p\left(i,j\right)$	${\tilde{p}}_{x}\left(i\right)$	$\sum_{j=1}^{N}\tilde{p}\left(i,j\right)\;\Delta$
*p*_*y*_(*j*)	$\sum_{i=1}^{N}p\left(i,j\right)$	${\tilde{p}}_{y}\left(j\right)$	$\sum_{i=1}^{N}\tilde{p}\left(i,j\right)\;\Delta$
*μ*_*x*_	$\sum_{i=1}^{N}i\cdot p_{x}\left(i\right)$	${\tilde{\mu }}_{x}$	$\sum_{i=1}^{N}\frac{i}{N}\cdot {\tilde{p}}_{x}\left(i\right)\;\Delta$
*μ*_*y*_	$\sum_{j=1}^{N}j\cdot p_{y}\left(j\right)$	${\tilde{\mu }}_{y}$	$\sum_{j=1}^{N}\frac{j}{N}\cdot {\tilde{p}}_{y}\left(j\right)\;\Delta$
$\sigma_{x}^{2}$	$\sum_{i=1}^{N}{\left(i-\mu_{x}\right)}^{2}\cdot p_{x}\left(i\right)$	${\tilde{\sigma }}_{x}^{2}$	$\sum_{i=1}^{N}{\left(\frac{i}{N}-{\tilde{\mu }}_{x}\right)}^{2}\cdot {\tilde{p}}_{x}\left(i\right)\;\Delta$
$\sigma_{y}^{2}$	$\sum_{j=1}^{N}{\left(j-\mu_{y}\right)}^{2}\cdot p_{y}\left(j\right)$	${\tilde{\sigma }}_{y}^{2}$	$\sum_{j=1}^{N}{\left(\frac{j}{N}-{\tilde{\mu }}_{y}\right)}^{2}\cdot {\tilde{p}}_{y}\left(j\right)\;\Delta$
*p*_*x*+*y*_(*k*)	${\sum_{i=1}^{N}\sum_{j=1}^{N}}_{i+j=k}p\left(i,j\right)$	${\tilde{p}}_{x+y}\left(k\right)$	${\sum_{i=1}^{N}\sum_{j=1}^{N}}_{i+j=k}\tilde{p}\left(i,j\right)\;\Delta$
*p*_*x*−*y*_(*k*)	${\sum_{i=1}^{N}\sum_{j=1}^{N}}_{|i-j|=k}p\left(i,j\right)$	${\tilde{p}}_{x-y}\left(k\right)$	${\sum_{i=1}^{N}\sum_{j=1}^{N}}_{|i-j|=k}\tilde{p}\left(i,j\right)\;\Delta$
*μ*_*x*+*y*_	$\sum_{k=2}^{2N}k\cdot p_{x+y}\left(k\right)$	${\tilde{\mu }}_{x+y}$	$\sum_{k=2}^{2N}\frac{2(k-1)}{2N-1}\cdot {\tilde{p}}_{x+y}\left(k\right)\;\Delta_{x+y}$
*μ*_*x*−*y*_	$\sum_{k=0}^{N-1}k\cdot p_{x-y}\left(k\right)$	${\tilde{\mu }}_{x-y}$	$\sum_{k=0}^{N-1}\frac{k+1}{N}\cdot {\tilde{p}}_{x-y}\left(k\right)\;\Delta$
*HX*	$-\sum_{i=1}^{N}p_{x}\left(i\right)\cdot \log p_{x}\left(i\right)$	$\tilde{HX}$	$-\sum_{i=1}^{N}{\tilde{p}}_{x}\left(i\right)\cdot \log{\tilde{p}}_{x}\left(i\right)\;\Delta$
*HY*	$-\sum_{i=1}^{N}p_{y}\left(i\right)\cdot \log p_{y}\left(i\right)$	$\tilde{HY}$	$-\sum_{i=1}^{N}{\tilde{p}}_{y}\left(j\right)\cdot \log{\tilde{p}}_{y}\left(j\right)\;\Delta$
*HXY*	$-\sum_{i=1}^{N}\sum_{j=1}^{N}p\left(i,j\right)\cdot \log p\left(i,j\right)$	$\tilde{HXY}$	$-\sum_{i=1}^{N}\sum_{j=1}^{N}\tilde{p}\left(i,j\right)\cdot \log\tilde{p}\left(i,j\right)\;\Delta_{ij}$
*HXY* 1	$-\sum_{i=1}^{N}\sum_{j=1}^{N}p\left(i,j\right)\cdot \log[p_{x}\left(i\right)\cdot p_{y}\left(j\right)]$	$\tilde{HXY1}$	$-\sum_{i=1}^{N}\sum_{j=1}^{N}\tilde{p}\left(i,j\right)\cdot \log[{\tilde{p}}_{x}\left(i\right)\cdot {\tilde{p}}_{y}\left(j\right)]\;\Delta_{ij}$
*HXY* 2	$-\sum_{i=1}^{N}\sum_{j=1}^{N}p_{x}\left(i\right)\cdot p_{y}\left(j\right)\cdot \log[p_{x}\left(i\right)\cdot p_{y}\left(j\right)]$	$\tilde{HXY2}$	$-\sum_{i=1}^{N}\sum_{j=1}^{N}{\tilde{p}}_{x}\left(i\right)\cdot {\tilde{p}}_{y}\left(j\right)\cdot \log[{\tilde{p}}_{x}\left(i\right)\cdot {\tilde{p}}_{y}\left(j\right)]\;\Delta_{ij}$
*Q*(*i*, *j*)	$\sum_{k=1}^{N}\frac{p(i,k)p(j,k)}{p_{x}\left(i\right)p_{y}\left(k\right)}$	$\tilde{Q}\left(i,j\right)$	$\sum_{k=1}^{N}\frac{\tilde{p}\left(i,k\right)\tilde{p}\left(j,k\right)}{{\tilde{p}}_{x}\left(i\right){\tilde{p}}_{y}\left(k\right)}\;\Delta_{ij}$

**Table 2:** The texture features computed from GLCMs. The middle column shows the original definitions, and the right column shows the modifications needed to make the features invariant to the number of gray-levels. There was an error in the definition of *Sum variance* in Haralick *et al*. [4], which has been corrected. λ_2_(*Q*(*i*, *j*)) denotes the second largest eigenvalue of a matrix *Q*(*i*, *j*). Note, however, that this feature is computationally unstable, and was therefore not included in the examples in this work. For symmetric GLCMs, *μ*_*x*_ and *μ*_*y*_ is identical, and is represented by *μ* in the expression for Cluster prominence and Cluster shade.

Feature	Original expression	Invariant expression
Autocorrelation [31]	$\sum_{i=1}^{N}\sum_{j=1}^{N}\left(i\cdot j\right)p(i,j)$	$\sum_{i=1}^{N}\sum_{j=1}^{N}(\frac{i}{N}\cdot \frac{j}{N})\tilde{p}(i,j)\;\Delta_{ij}$
Cluster prominence [4]	$\sum_{i=1}^{N}\sum_{j=1}^{N}{\left(i+j-2\mu \right)}^{3}p(i,j)$	$\sum_{i=1}^{N}\sum_{j=1}^{N}{\left(\frac{i}{N}+\frac{j}{N}-2\tilde{\mu }\right)}^{3}\tilde{p}(i,j)\;\Delta_{ij}$
Cluster shade [4]	$\sum_{i=1}^{N}\sum_{j=1}^{N}{\left(i+j-2\mu \right)}^{4}p(i,j)$	$\sum_{i=1}^{N}\sum_{j=1}^{N}{\left(\frac{i}{N}+\frac{j}{N}-2\tilde{\mu }\right)}^{4}\tilde{p}(i,j)\;\Delta_{ij}$
Contrast [4]	$\sum_{i=1}^{N}\sum_{j=1}^{N}{\left(i-j\right)}^{2}p(i,j)$	$\sum_{i=1}^{N}\sum_{j=1}^{N}{\left(\frac{i}{N}-\frac{j}{N}\right)}^{2}\tilde{p}(i,j)\;\Delta_{ij}$
Correlation [4]	$\sum_{i=1}^{N}\sum_{j=1}^{N}(\frac{i-\mu_{x}}{\sigma_{x}})(\frac{j-\mu_{y}}{\sigma_{y}})p\left(i,j\right)$	$\sum_{i=1}^{N}\sum_{j=1}^{N}(\frac{i/N-{\tilde{\mu }}_{x}}{{\tilde{\sigma }}_{x}})(\frac{j/N-{\tilde{\mu }}_{y}}{{\tilde{\sigma }}_{y}})\tilde{p}\left(i,j\right)\;\Delta_{ij}$
Difference entropy [4]	$-\sum_{k=0}^{N-1}p_{x-y}\left(k\right)\log p_{x-y}\left(k\right)$	$-\sum_{k=0}^{N-1}{\tilde{p}}_{x-y}\left(k\right)\log{\tilde{p}}_{x-y}\left(k\right)\;\Delta$
Difference variance [4]	$\sum_{k=0}^{N-1}{\left(k-\mu_{x-y}\right)}^{2}p_{x-y}(k)$	$\sum_{k=0}^{N-1}{\left(\frac{k+1}{N}-{\tilde{\mu }}_{x-y}\right)}^{2}{\tilde{p}}_{x-y}(k)\;\Delta$
Dissimilarity [31]	$\sum_{i=1}^{N}\sum_{j=1}^{N}|i-j|\cdot p\left(i,j\right)$	$\sum_{i=1}^{N}\sum_{j=1}^{N}|\frac{i}{N}-\frac{j}{N}|\cdot \tilde{p}\left(i,j\right)\;\Delta_{ij}$
Energy [4]	$\sum_{i=1}^{N}\sum_{j=1}^{N}p{\left(i,j\right)}^{2}$	$\sum_{i=1}^{N}\sum_{j=1}^{N}\tilde{p}{\left(i,j\right)}^{2}\;\Delta_{ij}$
Entropy [4]	$-\sum_{i=1}^{N}\sum_{j=1}^{N}p\left(i,j\right)\log p\left(i,j\right)$	$-\sum_{i=1}^{N}\sum_{j=1}^{N}\tilde{p}\left(i,j\right)\log\tilde{p}\left(i,j\right)\;\Delta_{ij}$
Homogeneity [31]	$\sum_{i=1}^{N}\sum_{j=1}^{N}\frac{p(i,j)}{1+{\left(i-j\right)}^{2}}$	$\sum_{i=1}^{N}\sum_{j=1}^{N}\frac{\tilde{p}\left(i,j\right)}{1+{\left(\frac{i}{N}-\frac{j}{N}\right)}^{2}}\;\Delta_{ij}$
Information measure of correlation 1 [4]	$\frac{HXY-HXY1}{\max(HX,HY)}$	$\frac{\tilde{HXY}-\tilde{HXY1}}{\max(\tilde{HX},\tilde{HY})}$
Information measure of correlation 2 [4]	$\sqrt{1-\exp[-2(HXY2-HXY)]}$	$\sqrt{1-\exp[-2(\tilde{HXY2}-\tilde{HXY})]}$
Inverse difference [38]	$\sum_{i=1}^{N}\sum_{j=1}^{N}\frac{p(i,j)}{1+|i-j|}$	$\sum_{i=1}^{N}\sum_{j=1}^{N}\frac{\tilde{p}\left(i,j\right)}{1+|\frac{i}{N}-\frac{j}{N}|}\;\Delta_{ij}$
Maximum probability [31]	$\max_{i,j}p\left(i,j\right)$	$\max_{i,j}\tilde{p}\left(i,j\right)$
Sum average [4], *μ*_*x*+*y*_	$\sum_{k=2}^{2N}kp_{x+y}\left(k\right)$	$\sum_{k=2}^{2N}\frac{2(k-1)}{2N-1}{\tilde{p}}_{x+y}\left(k\right)\;\Delta_{i+j}$
Sum entropy [4]	$-\sum_{k=2}^{2N}p_{x+y}\left(k\right)\log p_{x+y}\left(k\right)$	$-\sum_{k=2}^{2N}{\tilde{p}}_{x+y}\left(k\right)\log{\tilde{p}}_{x+y}\left(k\right)\;\Delta_{i+j}$
Sum of squares [4]	$\sum_{i=1}^{N}\sum_{j=1}^{N}{\left(i-\mu \right)}^{2}p\left(i,j\right)$	$\sum_{i=1}^{N}\sum_{j=1}^{N}{\left(\frac{i}{N}-\tilde{\mu }\right)}^{2}\tilde{p}\left(i,j\right)\;\Delta_{ij}$
Sum variance [4]	$\sum_{k=2}^{2N}{\left(k-\mu_{x+y}\right)}^{2}p_{x+y}\left(k\right)$	$\sum_{k=2}^{2N}{\left(\frac{2(k-1)}{2N-1}-{\tilde{\mu }}_{x+y}\right)}^{2}{\tilde{p}}_{x+y}\left(k\right)\;\Delta_{i+j}$
Maximal Correlation Coefficient [4]	$\sqrt{\lambda_{2}(Q(i,j))}$	$\sqrt{\lambda_{2}(\tilde{Q}\left(i,j\right))}$

**Fig 1:**
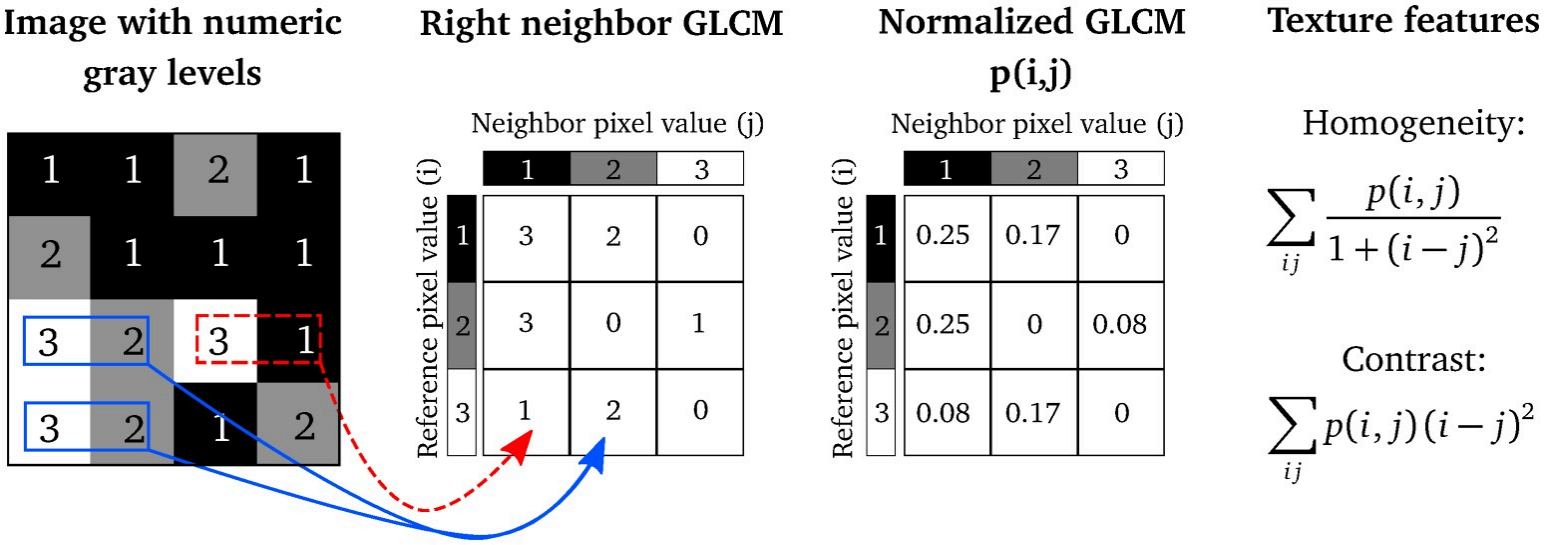
An illustration of how the Haralick texture features are computed. In a 4 × 4 image, three gray-levels are represented by numerical values from 1 to 3. The GLCM is constructed by considering the relation of each voxel with its neighborhood. In this example, for simplicity, we only look at the neighbor to the right. The GLCM acts like a counter for every combination of gray-level pairs in the image. For each voxel, its value and the neighboring voxel value are counted in a specific GLCM element. The value of the reference voxel determines the column of the GLCM and the neighbor value determines the row. In this ROI, there are two instances when a reference voxel of 3 “co-occurs” with a neighbor voxel of 2 (indicated in solid, blue), and there is one instance of a reference voxel of 3 with a neighbor voxel of 1 (indicated in dashed, red). The normalized GLCM represents the estimated probability of each combination to occur in the image. The Haralick texture features are functions of the normalized GLCM, where different aspects of the gray-level distribution in the ROI are represented. For example, diagonal elements in the GLCM represent voxels pairs with equal gray-levels. The texture feature “contrast” gives elements with similar gray-level values a low weight but elements with dissimilar gray-levels a high weight. Reprinted from Brynolfsson *et al*. [37] under a CC BY license, with permission from Nature Publishing Group, original copyright 2017.

Fig 1 illustrates Steps 2–4 of computing the Haralick texture features from a 4 × 4 example image, that has already been quantized to three gray-levels.

The number of gray-levels in the quantized image determines the size of the GLCM, and will affect the texture feature values. By increasing the number of gray-levels, *N*, the sums in Tables 1 and 2 will change. Further, the element values, *p*(*i*, *j*), of the normalized GLCM will decrease as the number of gray-levels increases, since the sum of all elements is unity. These two properties of the GLCM and the Haralick texture features means that the texture feature values depend on the number of gray-levels in the quantized image.

### Invariant texture features

To develop asymptotically invariant texture features, we propose an interpretation of the GLCM as a discrete approximation of a probability density function. In this interpretation, the texture features are expressed as integrals over functions of the density, *i.e*. as

$$\begin{array}{c} \int_{0}^{1}\int_{0}^{1}\phi \left(i,j,g\left(p^{*}\right)\right)\psi \left(p^{*}\left(i,j\right)\right)\;di\;dj\;, \end{array}$$ (7)

where p* is the underlying *true* probability density function, for which

$$\begin{array}{c} \int_{0}^{1}\int_{0}^{1}p^{*}\left(i,j\right)\;di\;dj=1\;. \end{array}$$ (8)

**Fig 2:**
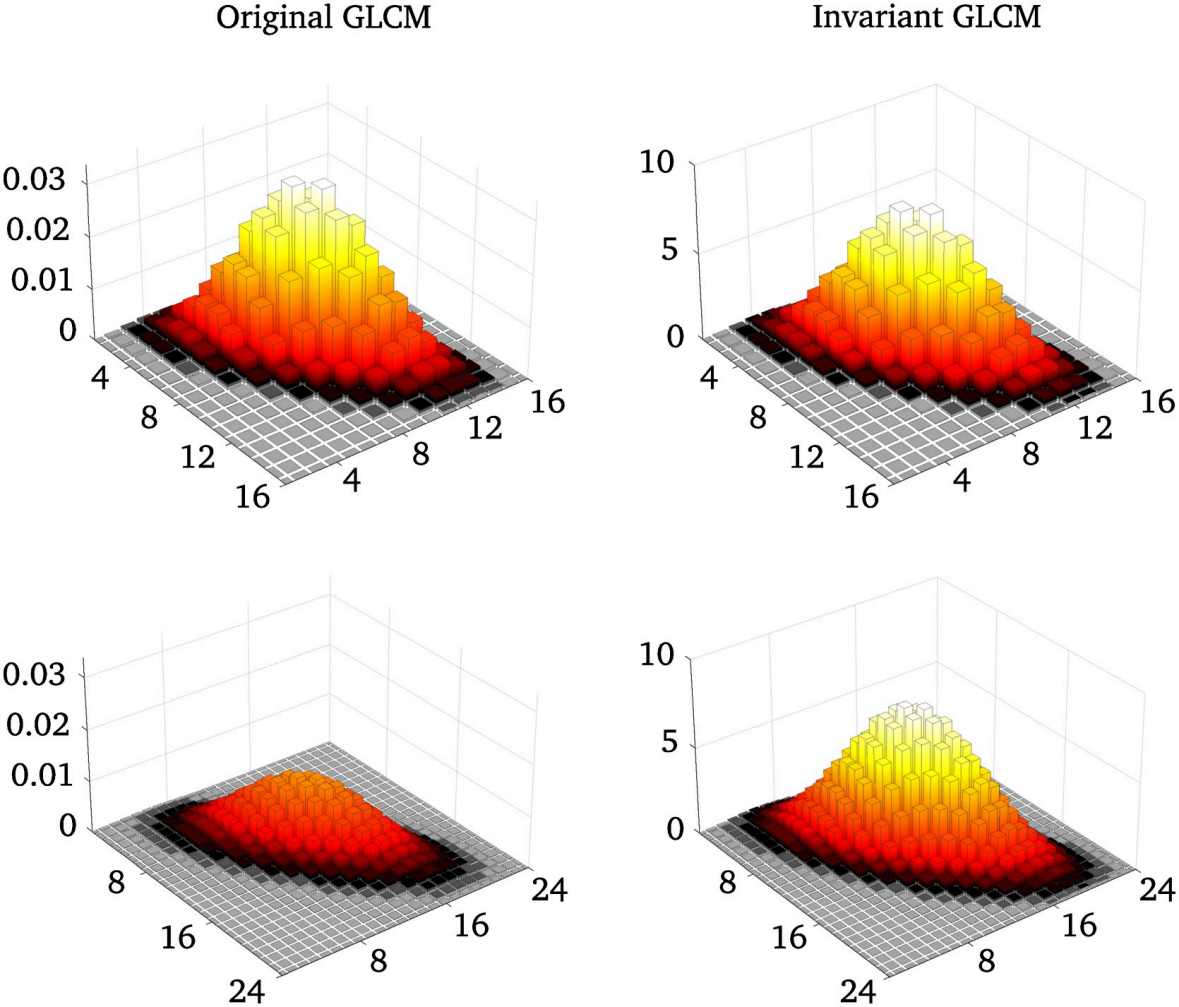
A comparison between a original GLCM and an invariant GLCM for different numbers of bins. Synthetic GLCMs generated as bivariate Gaussian distributions, for two different quantization levels, 16 and 24 gray-levels. The elements of the standard GLCM sums to 1, which means that the GLCM element values will decrease with increasing GLCM size. The invariant GLCMs are normalized to keep the total volume of the GLCM at 1, where each element is ascribed an area of 1/*N*^2^ units.

Eq 7 can be approximated by a Riemann sum:

$$\begin{array}{c} \int_{0}^{1}\int_{0}^{1}\phi (i,j,g(p^{*}))\psi (p^{*}\left(i,j\right))\;di\;dj\approx \sum_{i=1}^{N}\sum_{j=1}^{N}\phi (\frac{i}{N},\frac{j}{N},g\left(\tilde{P}\right))\psi (\tilde{p}\left(i,j\right))\Delta_{i}\Delta_{j}\;, \end{array}$$ (9)

where

$$\begin{array}{c} \Delta_{i}=\Delta_{j}=\frac{1}{N}\;, \end{array}$$ (10)

and

$$\begin{array}{c} \tilde{P}=\frac{X}{\sum_{i=1}^{N}\sum_{j=1}^{N}X\left(i,j\right)\Delta_{i}\Delta_{j}}\;, \end{array}$$ (11)

where Δ_*i*_ and Δ_*j*_ are differentials, and $\tilde{P}$ is the invariant GLCM. So, to make the Haralick texture features invariant to the number of gray-levels, *i* and *j* are normalized to the half-open interval (0, 1], the sums are multiplied by differentials, and the GLCM is normalized so that its Riemann sum is 1, see Fig 2 and Eq 11. The proposed invariant texture features can be seen in Columns 3 and 4 of Tables 1 and 2. The discrete approximations of the integrals depend on the discretization of the distribution (*i.e*., the quantization, or the number of gray-levels), but the approximation is equal to the integral in the limit as *N* → ∞. The original features do not give the same approximated values for different discretizations, they either approach zero, a fixed value or infinity when *N* → ∞.

## Materials and methods

**Fig 3:**
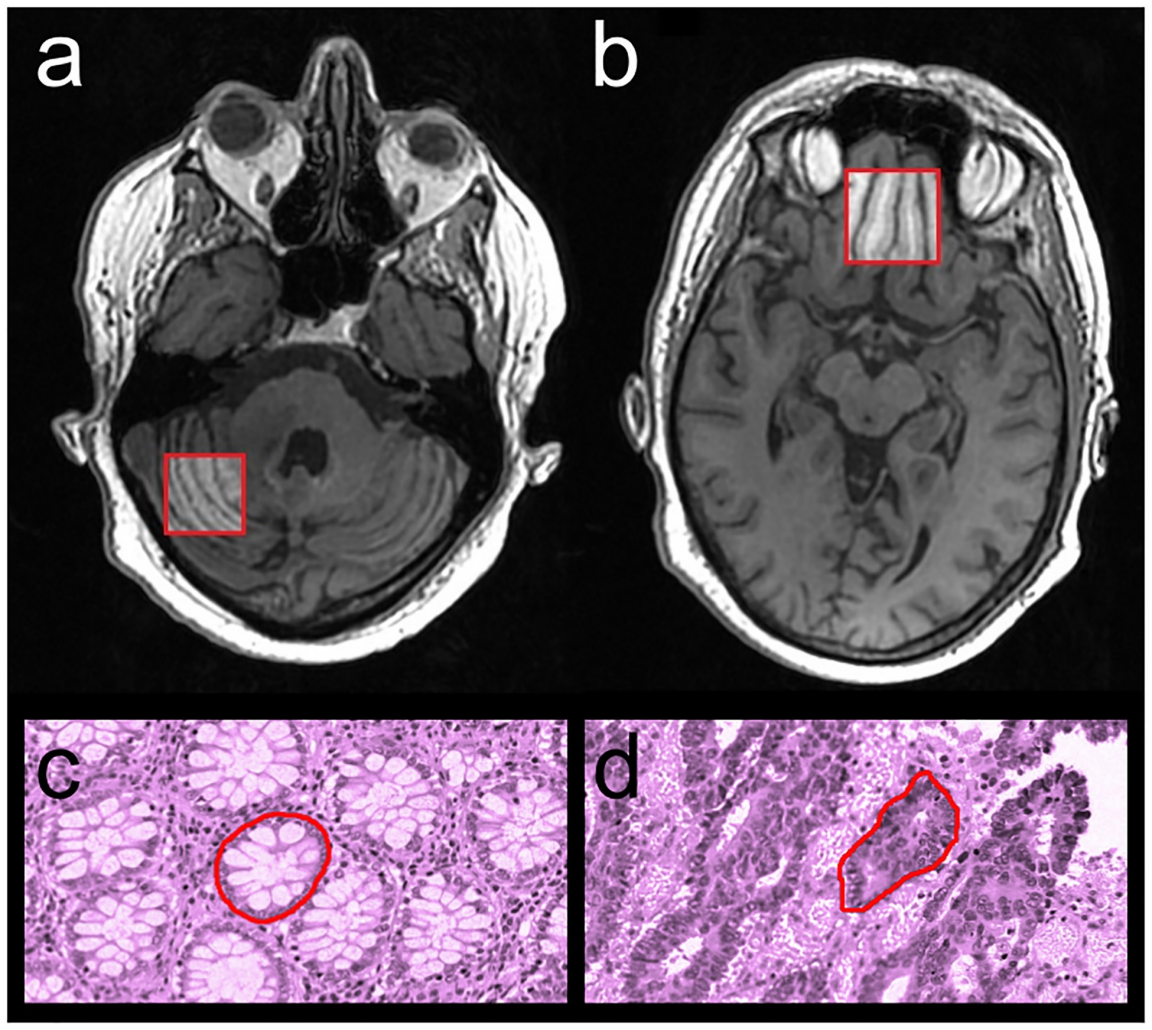
The image data used to test the invariant features in classification problems. Texture features were computed for a range of quantization gray-levels from a region in the cerebellum (a) and a region in the prefrontal cortex (b), and from benign (c) and malignant (d) colorectal glandular structures.

We investigated the properties of the original and invariant texture features using two data sets. Dataset 1 contained T1-weighted MRI volumes of the brain. We computed texture features for the cerebellum and the prefrontal cortex, see Fig 3. We used logistic regression to classify each region based on their texture properties, and compared the result for the two methods. This dataset has limited clinical utility, and is used to compare the performance of the original and invariant texture features only. Dataset 2 contained histology images of colorectal cancer glands from the Warwick-QU dataset [40, 41]. This is an open dataset that was used in the Gland Segmentation in Colon Histology Images Challenge Contest (GlaS), held at MICCAI’2015. We computed texture features for each gland, and used logistic regression to classify each gland as benign or malignant.

### Dataset 1 imaging

Dataset 1 contained 81 T1-weighted axial spoiled gradient echo images of 29 subjects. The image field of view was 250 × 250 × 240 mm^3^, with an in-plane resolution of 1.3 × 1.3 mm^2^ and a slice thickness of 1 mm. The repetition time and echo time were 7.1 and 2.5 ms, respectively, and the bandwidth was 392 Hz/pixel. A region of the prefrontal cortex (30 × 30 × 10 mm^3^, 23 × 23 × 10 voxels), and a region of the cerebellum (25 × 25 × 10 mm^3^, 19 × 19 × 10 voxels), were delineated on a reference image set, see Fig 3. The other images were rigidly registered to the reference, and the corresponding regions were extracted. Twenty-three images from 13 patients where the image registration failed were excluded from the study. The clinical trial was approved by the local Regional Ethical Review Board of Umeå University, and oral and written consent was given by all subjects.

### Dataset 2 imaging

Dataset 2 contained 1,518 images of colorectal glandular structures in 165 Hematoxylin and Eosin (H&E) stained slides. The slides were digitally scanned at 20 × magnification, with a resolution of 0.62005 *μ*m/pixel, using a Zeiss MIRAX MIDI Slide Scanner. The image dimensions were 520 × 775 pixels for 151 slide scans, and 430 × 575 pixels for 14 slides. 934 gland structures in 74 slides were classified as benign and 584 structures in 91 slides as malignant. The luminance, calculated from the RGB slides were used in the analysis. The images were dithered using uniform noise, to reduce quantization errors.

### Texture analysis

Thirty-two direction invariant GLCMs were created from the immediate voxel neighbors in Eq 4, for each brain region or gland structure, one for every quantization of 8–256 gray-levels in steps of 8. We used fixed upper and lower limits of 1500 and 4500 pixel units for the cerebellum and 1000 and 8000 pixel units for the prefrontal cortex, determined by the limits of the histograms from all regions. The upper and lower limits of the glandular structures were set to 0–256. The 20 original Haralick features and the corresponding invariant features described in Table 2 were computed for each quantization. All texture analysis was done using MICE Toolkit [42] and MATLAB 2016b (MathWorks, Inc., Natick, MA).

### Classification

The goal of each logistic regression model was to correctly predict the brain regions (cerebellum or prefrontal cortex) in Dataset 1 or the glandular structures (benign or malignant) in Dataset 2, from the texture values. Two scenarios were explored. In the first scenario, the datasets were split into training (50%) and test dataset (50%). One hundred classifiers were trained using a random quantization of each member in the training data. These models were used to predict the correct class in the test set for all quantization levels. In the second scenario, the datasets were split into training (50%), validation (25%) and test (25%) sets. One classifier was trained for each of the 32 quantizations. These classifiers were used to predict the correct class in the test set for all quantization levels. For each dataset and texture method, the validation set was used to perform variable selection to remove irrelevant or highly correlated features. We used forward selection [43], and maximized the mean accuracy.

### Evaluation

The first scenario was evaluated by the average accuracy of the 100 classifiers that was trained on randomly quantized data. We compared the original and invariant Haralick features using a two-sample, two-tailed Welch’s *t*-test for populations with unequal variances. The second scenario was evaluated using a Mann-Whitney *U*-test to compare the results of the original and invariant Haralick features.

## Results

**Fig 4:**
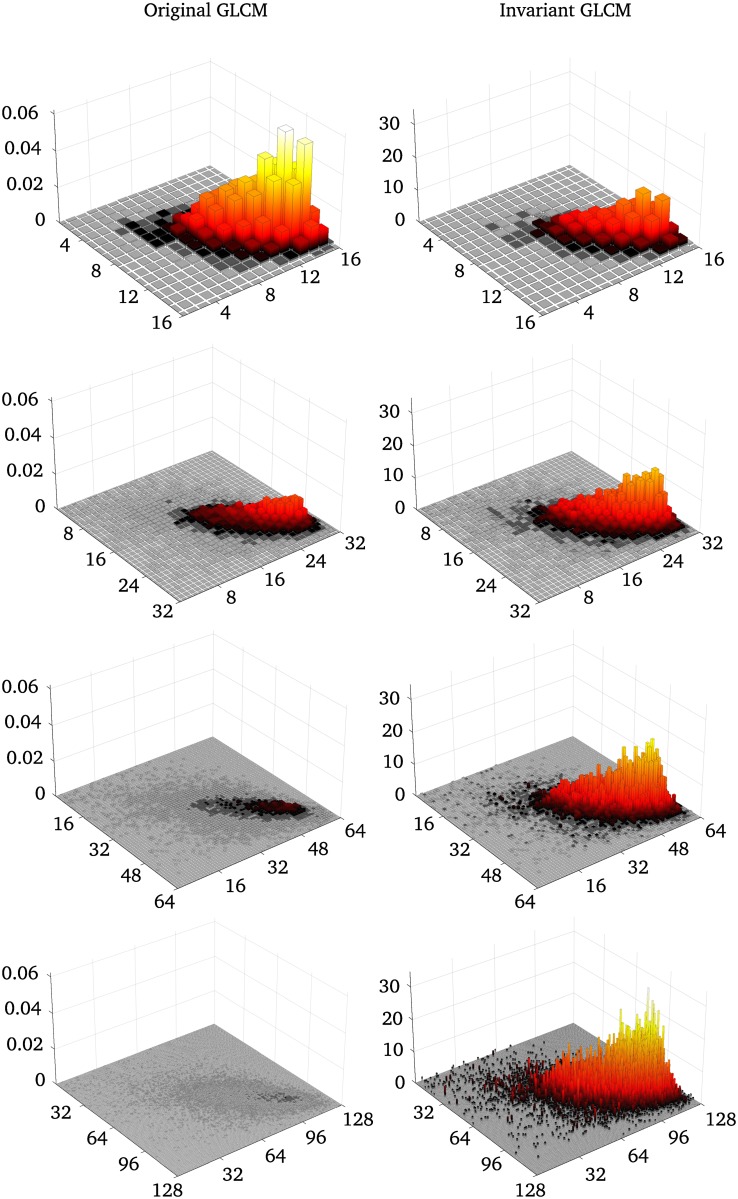
Example of original and invariant GLCMs for different quantization levels from Dataset 1. The left column shows the original GLCMs created from the cerebellum region in Fig 3, for different quantization levels. The right column shows the invariant GLCMs for the same quantization levels. The original GLCMs are normalized so that the sum is 1, whereas the invariant features are normalized so that the volume of the GLCM is 1.

Fig 4 shows the original GLCMs (left) and the invariant GLCMs (right) for the reference region of the cerebellum for 16, 32, 64, and 128 gray-levels. The standard GLCMs decrease in amplitude with orders of magnitude as the number of gray-levels increase. The invariant features retain the same volume, and the amplitude only increases slightly between 8 and 128 gray-levels, due to noise introduced with increased gray-level resolution.

**Fig 5:**
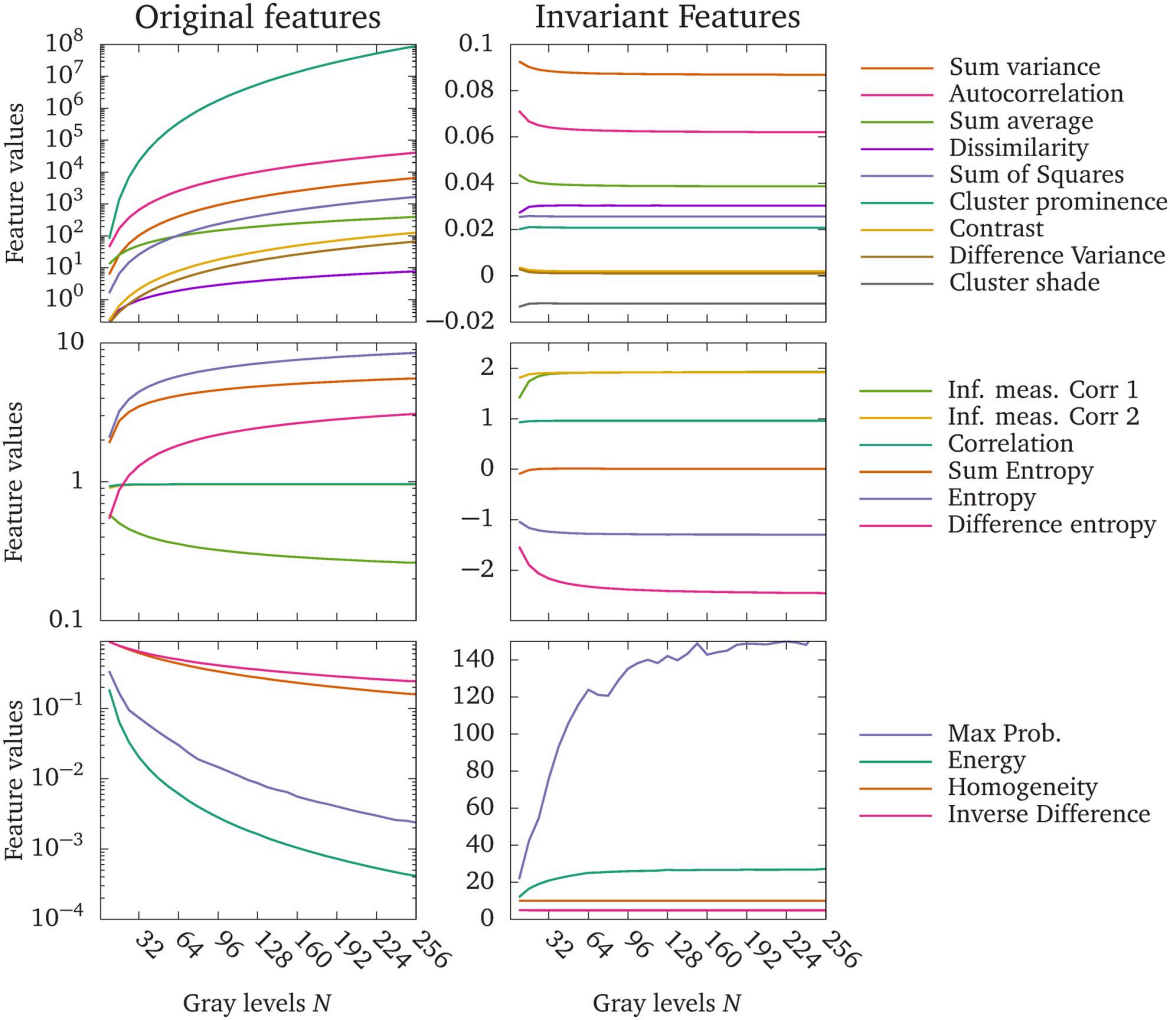
The original Haralick feature values, and the proposed invariant feature values. Texture feature values computed from a benign glandular structure in the gland dataset. The upper left plot shows the original Haralick features that increase rapidly with the number of gray-levels. The middle left plot shows original Haralick features that increase modestly, or reach a plateau with increasing number of gray-levels. The bottom left plot shows original Haralick features that decrease with increasing number of gray-levels. The right column shows the corresponding invariant features. Note that the original features are plotted on a log-scale to accommodate the wide range of values. The *Information measure of Correlation 2* and *Correlation* overlap in the original features. The *Information Measure of Correlation 1* is negative in the original features, but the absolute values are displayed in the graph to accommodate the log scale.

Fig 5 shows how the texture feature values change with the number of gray-levels of the GLCM, computed from a benign glandular structure in the gland dataset (Dataset 2). In the left column, the upper graph shows features that increase rapidly with the number of gray-levels, the middle graph shows features that increase modestly, or reach a limit as the number of gray-levels increases, and the lower graph shows features that decrease with increasing number of gray-levels. The right column shows the corresponding invariant texture features. Note that the original features are plotted on a logarithmic scale on the vertical axis, whereas the invariant features are plotted on a linear scale.

**Fig 6:**
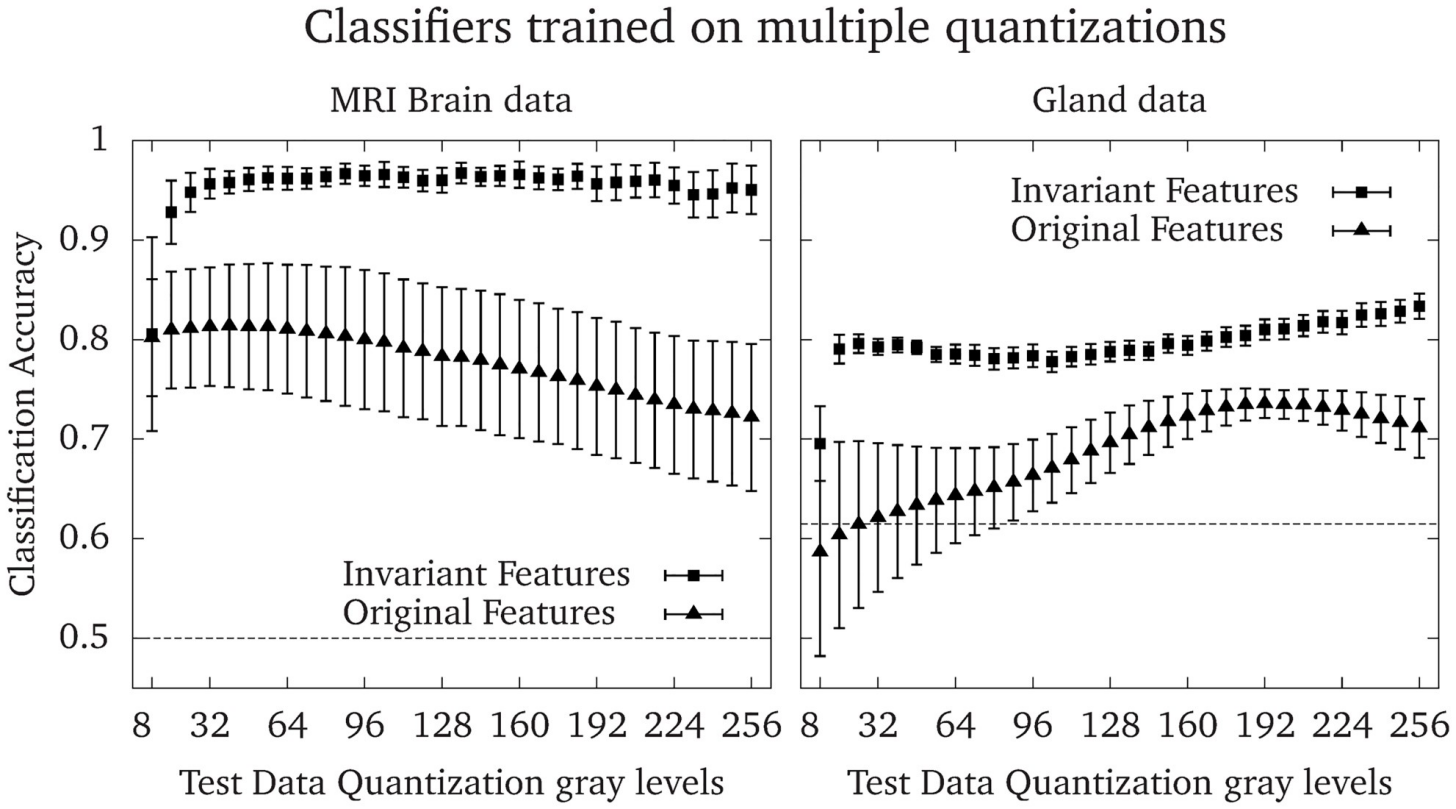
Accuracy of classifiers trained on multiple quantization gray-levels. To evaluate the performance of classifiers that were trained on a wide range of quantizations, 100 logistic regression models were trained for each data set and method. Each image in the training data was randomly quantized to one of 32 quantization gray-levels between 8 and 256 for every model. The markers show the mean accuracy and the error bars show the standard deviations of the accuracy for each quantization of the test data. The dashed line shows the accuracy obtained by assigning all predictions to the most common class, which is 0.5 for either the cerebellum or prefrontal cortex in Dataset 1 and 0.615 for benign glandular structures in Dataset 2.

Fig 6 shows the accuracy of 100 logistic regression models trained on randomly quantized images in the range of 8–256 gray-levels in steps of 8, and tested on images quantized to all gray-levels in the range 8–256 in steps of 8. The error bars show the standard deviation of the accuracy of the 100 classifiers, for each test dataset quantization level. Consistently, classifiers trained on the invariant features outperformed classifiers trained on the original features, and had a lower standard deviation of the accuracy. The average accuracies were 0.77 ± 0.08 and 0.96 ± 0.03 (*p* < 10^−39^, *t* = 20.82, using a two-sample Welch’s t-test with 138 degrees of freedom, estimated using the Satterthwaite-Welch formula [44]) for Dataset 1 (the brain dataset), and 0.69 ± 0.06 and 0.80 ± 0.03 (*p* < 10^−35^, *t* = 17.15, using a two-sample Welch’s t-test with 142 degrees of freedom [44]) for Dataset 2 (the gland dataset) for the original and invariant features, respectively.

**Fig 7:**
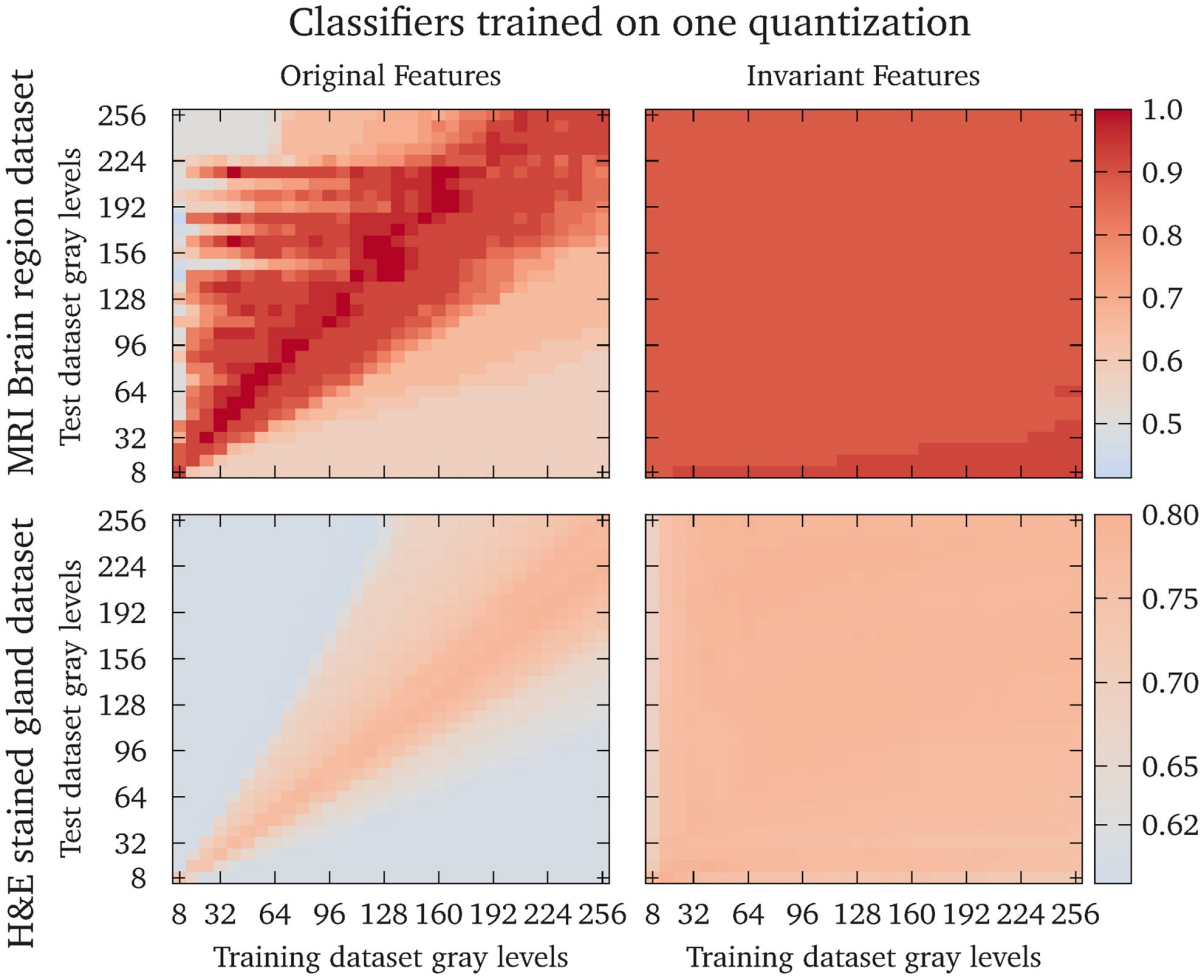
Accuracy of classifiers trained on one quantization gray-level. The heatmaps show the accuracy of logistic regression models trained on one quantization gray-level and tested on all quantization gray-levels in the range 8–256 in steps of 8. The colormap is scaled to show a neutral gray for the accuracy obtained by assigning all predictions to the most common class, which is 0.5 for either the cerebellum or prefrontal cortex in Dataset 1 and 0.615 for benign glandular structures in Dataset 2. The upper row shows the result from Dataset 1 and the lower row shows the results from Dataset 2. The left column shows the original features and the right column shows the invariant features. The diagonal elements show the accuracies where the same quantization gray-levels were used for the training and test data.

Fig 7 shows the accuracy of the logistic regression models trained on one quantization levels, and tested on all quantization levels in the range 8–256 in steps of 8, for Dataset 1 (the brain dataset) and Dataset 2 (the gland dataset). The classifiers that were trained and tested on the original features had a high accuracy only when the test data quantization was close to the training data quantization. The classifiers that were trained on the invariant features had a high accuracy for all combinations of training and test dataset quantizations. The renormalized feature accuracies were significantly larger (*p* < 10^−99^ using a Mann-Whitney U-test) for both datasets.

## Discussion

We have presented a simple modification of the Haralick texture features that makes them asymptotically invariant to the numbers of gray-levels in the quantizations. This is achieved by viewing the GLCM as a discrete approximation of a probability density function, instead of a probability mass function, over the pairs of gray-levels in the image. We have shown examples of how the standard and invariant GLCMs scale with increasing bit depth, Fig 4, and how most of the modified texture features quickly approach a limit, whereas most of the original features diverge or converge to zero, Fig 5. We have demonstrated the benefit of the proposed modified features by training logistic regression models to separate two brain regions in Dataset 1, and the malignancy of colorectal glands in Dataset 2, based only on texture features. Classifiers based on the invariant features performed better than the original features in all tests performed in this work. Further, the invariant Haralick features are rescaled versions of the original features, which means that they retain their original interpretations in most cases. Finally, the proposed modifications allow texture features to be reproducible regardless of quantization, since the same texture feature will give similar values independent of the quantization.

We tested the texture features in two scenarios. In the first scenario, we trained classifiers on texture features computed from images with different quantizations. In this scenario, the quantization levels might be chosen to enhance features optimally in each image, which is employed by *e.g*. Leijenenaar *et al*. [45]. Fig 6 shows that models trained on the invariant features have a higher accuracy and smaller standard deviation than models trained on the original features. In the second scenario, we trained the classifiers on one quantization level and predicted the classes from features computed on each of the other quantization levels. This scenario represents cases in *e.g*. radiomics, where a predictive model is created using one quantization, and employed to other datasets where the quantization is different due to *e.g*. resolution constrains, the size of the ROI, or where the optimal quantization is different. The classifiers trained on the invariant features had equal or higher accuracies compared to classifiers trained on the original features for the same combination of training and test dataset quantizations in most cases. The exceptions were some classifiers trained on the original features where the test quantization is slightly higher than the training quantization, see the upper left plot in Fig 7. In these situations, feature differences between the two classes are enhanced by the changes in feature values of the test data due to the different quantization gray-levels, as shown in Fig 5. This effect depends on the inherent texture of the two classes to be separated, and the features that are used in the model.

*Maximum probability* is very sensitive to noise, which is evident from Figs 4 and 5. For the invariant features, the *maximum probability* is now interpreted as the *maximum probability density*, *i.e*. the density value at the mode.

In the continuous case with the invariant features, the entropy becomes a differential entropy, and no longer has all the properties of the discrete counterpart. For instance, the differential entropy can be negative. This reinterpretation has particularly problematic consequences for the *Information Measure of Correlation 1*, (see Table 2). The denominator can be both positive and negative, and in particular, it can be close to zero. We note that in our particular case, with integration limits running from zero to one, division by zero can only occur if either of the marginal distributions of **P** are uniform.

It is important to note that except for maximum probability and the features that involve entropy, the interpretations of the texture features do not change. The invariant features are obtained by renormalizing the GLCM, the indices, and by multiplication with a differential determined by the bin size. It is merely a rescaling of the features to make them independent of the number of gray-levels of the GLCM. This has generally no impact on the interpretations of these features.

We chose to study the behavior of the invariant features between 8 and 256 quantization gray-levels. Too few gray levels will lead to a crude Riemann approximation in Eq 9, which can be seen in *e.g*. Fig 5 where the feature values stabilize around 16 gray-levels for most features, or in Figs 6 and 7 where the accuracies improve drastically between 8 and 16 gray-levels. Choosing excessively many quantizations gray-levels, *e.g*. on the order of the pixels present in the image, can produce sparse GLCMs. The invariant features will suffer from the same symptoms as the original features in this regard, *i.e*. an over-sensitivity to noise; and for small image regions which will produce sparse GLCMs, a failure to properly represent the underlying texture information.

Clausi *et al*. [38] proposed a normalized version of Homogeneity and Inverse difference by dividing the indices *i* and *j* by *N* to improve classification. The result is equivalent to the invariant versions presented in Table 2, since the effect of the differential Δ_*ij*_ and the renormalized GLCM, $\tilde{p}\left(i,j\right)$ cancel out in these features. However Clausi’s approach to only normalize the indices will not render all features invariant to the quantization; features such as Energy, Entropy measures, Variance measures, Information measure of correlation 1 and 2 and Maximum probability are not explicitly expressed in terms of the GLCM indices and cannot be normalized in this way. Shafic-ul-Hassan *et al*. [39] empirically derive normalization factors in terms of the quantization gray-levels for a selection of Haralick features. Of those, only one (Contrast) were identical to our results. A few of their normalized features were similar but not identical to the results presented here. Hence, most empirical derivations presented by Shafic-ul-Hassan do not fit the theory presented in this work. Further, many features presented in Table 2 cannot be reduced to a simple scaling factor of the original features, *e.g*. any feature containing entropy.

In this study we chose global min-max limits when quantizing the images in each dataset to minimize variations in the feature values due to image noise or other structures in the region of interest. Another approach is to set the limits for each ROI based on the minimum and maximum values inside each ROI [37, 45]. However, one extremely high or low pixel value inside the ROI will shift the center of mass of the GLCM, drastically affecting the Haralick feature values. The global limits approach requires that the image intensities are comparable between all images in the dataset, which requires that the images are acquired with the same hardware and imaging settings, or it can be achieved by *e.g*. normalizing the intensity of common structures in the images.

The invariant features can be used when analyzing multiple images of different sizes (different number of pixels), and possibly even different amounts of noise, by optimizing the quantization level for each image, while still obtaining comparable texture features. Image noise will affect the texture feature values [36, 37, 46], but the smoothing effect of gray-level quantization could reduce the impact on the resulting features. Some features are more sensitive to noise [37], and a more aggressive gray-level quantization for the invariant version of the features could reduce impact of the noise, while retaining the possibility to compare feature values with those from images analyzed at different quantization levels. This approach requires further research. Finally, there are other methods of extracting features, such as the Gray-level Size Zone Matrix (GLSZM), Gray-level Run Length Matrix (GLRLM) and the Neighborhood Gray-tone Difference Matrix (NGTDM), which feature values are also affected by the gray-level quantization. Shafic-ul-Hassan *et al*. [39] proposes empirical normalization factors for some features from these methods, and it is be feasible that a similar approach to what is presented in this work could reduce the impact of gray-level quantization to these methods as well. Making more methods independent of the gray-level quantization will increase the applicability and reproducibility of radiomics analyses, so this is also an interesting prospect for future research.

## Conclusion

By reinterpreting the GLCM as a discretized probability density function, it is possible to construct a modified set of Haralick texture features that are asymptotically invariant to the image quantization. Except for maximum probability and features involving entropy, the invariant features retain their original interpretations. We show that the invariant features can be used in different classification setups, with results superior to the original definitions. This mean that the invariant Haralick texture features are reproducible even when different gray-level quantizations are used, unlike the original definitions.
